# Correction to ‘Gotree/Goalign: toolkit and Go API to facilitate the development of phylogenetic workflows’

**DOI:** 10.1093/nargab/lqab083

**Published:** 2021-09-10

**Authors:** Frédéric Lemoine, Olivier Gascuel

**Affiliations:** Unité de Bioinformatique Evolutive, Département de Biologie Computationnelle, Institut Pasteur, Paris, France; Hub de Bioinformatique et Biostatistique, Département de Biologie Computationnelle, Institut Pasteur, Paris, France; Unité de Bioinformatique Evolutive, Département de Biologie Computationnelle, Institut Pasteur, Paris, France; Institut de Systématique, Evolution, Biodiversité, ISYEB, UMR 7205, Muséum National d’Histoire Naturelle, CNRS, SU, EPHE, UA, Paris, France

The correct affiliations for Olivier Gascuel in the above article are as follows:

^1^Unité de Bioinformatique Evolutive, Département de Biologie Computationnelle, Institut Pasteur, Paris, France

^3^Institut de Systématique, Evolution, Biodiversité, ISYEB, UMR 7205, Muséum National d’Histoire Naturelle, CNRS, SU, EPHE, UA, Paris, France

The published article (1) has been updated.
