# Supplementary material for: Gotree/Goalign: toolkit and Go API to facilitate the development of phylogenetic workflows
Source: NAR Genom Bioinform. 2021 Aug 11;3(3):lqab075. doi: 10.1093/nargab/lqab075 (PMC8356961; doi:10.1093/nargab/lqab075)
Supplement: lqab075_Supplemental_File [file lqab075_supplemental_file.pdf]

SUPPLEMENTARY INFORMATION

Gotree/Goalign : Toolkit and Go API to facilitate the development of phylogenetic workflows

Frédéric Lemoine[1,2]* and Olivier Gascuel[1,3]

1 Unité de Bioinformatique Évolutive, Département de Biologie Computationnelle, Institut Pasteur, Paris, FRANCE,
2 Hub de Bioinformatique et Biostatistique, Département de Biologie Computationnelle, Institut Pasteur, Paris, FRANCE,
3 Current address: Institut de Systématique, Evolution, Biodiversité, ISYEB, UMR 7205, Muséum National d'Histoire Naturelle, CNRS, SU, EPHE, UA, Paris, FRANCE

*To whom correspondence should be addressed: frederic.lemoine@pasteur.fr

---

Supp. Text 1: Examples of Gotree/Goalign commands **pp. 2-4**

Supp. Figure S1: Representation of the use case workflow and command templates **pp. 5-6**

Supp. Data 1: Nextflow implementation of the use case **pp. 7-8**

Supp. Data 2: List of analyzed primate species **pp. 9**

Supp. Data 3: List of 1,315 orthologous groups from OrthoDB **pp. 10-15**

Supplementary Text 1: Examples of Gotree/Goalign commands

The comprehensive list of Gotree/Goalign commands is given on their respective GitHub repositories:
https://github.com/evolbioinfo/gotree/blob/master/docs/index.md
https://github.com/evolbioinfo/goalign/blob/master/docs/index.md

1) Reformatting a tree from newick to nexus[1]

```
gotree reformat nexus -i itol://1292153021730731119304816660
```

The input tree is directly downloaded from iTOL, using its identifier and reformatted in Newick locally.

2) Reformatting an alignment from Fasta to Phylip[1]

```
goalign reformat phylip -i https://github.com/evolbioinfo/goalign/raw/master/tests/data/test_xz.xz
```

The input alignment is automatically downloaded from a remote server, and locally reformatted to Phylip.

3) Display basic summary statistics of a tree from TreeBase[2] :

```
gotree stats --format nexus -i treebase://Tr61953
```

The input tree is directly downloaded from TreeBase, and the following summary statistics are displayed: the number of nodes, tips and edges, the average and total branch length, the average and median support, the number of cherries, and the Colless and Sackin tree balance indices (if rooted).

4) Computing basic summary statistics of a tree from iTOL[2]

```
gotree stats -i itol://1292153021730731119304816660
```

This performs the same operation as the previous command, but after downloading the input tree from iTOL.

5) Computing basic summary statistics on a remote alignment[3]

```
goalign stats -i https://github.com/evolbioinfo/goalign/raw/master/tests/data/test_xz.xz
```

The input alignment is downloaded from a remote server, and the following summary statistics are displayed: the length of the alignment, the number of sequences, the average number of different characters per site, the number of variable sites, and the number of occurrences and the frequency of each character (nucleotide or amino-acid).

6) Drawing a tree in the console[4]

```
gotree draw text -w 100 -i
   https://github.com/evolbioinfo/gotree/raw/master/tests/data/rand_tree.nw.gz
```

The input tree is downloaded from a remote server, and the tree is displayed in the console in Phylip like text mode. For example:

```
+ Tip4
|
|---- Tip0
|
|                 +--------- Tip5
|              +--|
|              |  |+--------------------- Tip6
|              |  +|
|              |   |       +------ Tip9
|           +--|   +-------|
|           |  |           +--------------------- Tip3
|           |  |
|           |  |   +- Tip7
+-----------|  +---|
            |      |                        + Tip8
            |      +------------------------|
            |                               +-- Tip2
            |
            +---------------------- Tip1
```

1 https://github.com/evolbioinfo/gotree/blob/master/docs/commands/reformat.md
2 https://github.com/evolbioinfo/gotree/blob/master/docs/commands/stats.md
3 https://github.com/evolbioinfo/goalign/blob/master/docs/commands/stats.md
4 https://github.com/evolbioinfo/goalign/blob/master/docs/commands/draw.md

7) Rerooting a tree[5]

```
gotree reroot outgroup -i
   https://github.com/evolbioinfo/gotree/raw/master/tests/data/rand_tree.nw.gz Tip484 Tip410 Tip36
```

The input tree is downloaded from a remote server, and is rerooted using the given outgroup defined by a set of tips.

8) Collapsing short branches from a tree[6]

```
gotree collapse length -l 0.01 -i
   https://github.com/evolbioinfo/gotree/raw/master/tests/data/rand_tree.nw.gz
```

The tree is downloaded from a remote server, and branches that are shorter than 0.01 are collapsed, producing polytomies.

9) Compute patristic distance matrix[7]

```
gotree matrix -i https://github.com/evolbioinfo/gotree/raw/master/tests/data/rand_tree.nw.gz
```

The tree is downloaded from a remote server, and the patristic distance matrix is computed (summing over the branch lengths along the paths between all pairs of tips).

10) Mask sites from an alignment[8]

```
goalign mask -s 3 -l 10 -i https://github.com/evolbioinfo/goalign/raw/master/tests/data/test_xz.xz
```

The alignment is downloaded from a remote server, and 10 sites from the 4th one (indices start at 0) are masked (replaced by Ns or Xs).

11) Filter out sequences from an alignment[9]

```
goalign subset -r -i https://github.com/evolbioinfo/goalign/raw/master/tests/data/test_xz.xz Seq0002
   Seq0003
```

The input alignment is downloaded from a remote server, and all sequences are removed except the ones given in the command line.

12) Filter out sites from an alignment[10]

```
goalign subsites -r -i https://github.com/evolbioinfo/goalign/raw/master/tests/data/test_xz.xz 1 2 3
```

The input alignment is downloaded from a remote server, and all sites are removed except the ones with indices given in the command line.

13) Compute distances between sequences[11]

```
goalign compute distance -i
   https://github.com/evolbioinfo/goalign/raw/master/tests/data/test_distance.phy.gz --phylip -m jc
```

The DNA input alignment is downloaded from a remote server (--phylip is given because the input format is phylip) and the distance matrix is computed using Jukes and Cantor (1969) evolutionary model (it can be pdist, JC, K2P, F81, F84 and TN93 for DNA alignments, and DAYHOFF, JTT, MtRev, LG and WAG for protein alignments).

14) Concatenate several alignments (merging sequences coming from the same species/taxa)[12]

```
goalign concat -i https://github.com/evolbioinfo/goalign/raw/master/tests/data/test_xz.xz
```

[5] https://github.com/evolbioinfo/gotree/blob/master/docs/commands/reroot.md
[6] https://github.com/evolbioinfo/gotree/blob/master/docs/commands/collapse.md
[7] https://github.com/evolbioinfo/gotree/blob/master/docs/commands/matrix.md
[8] https://github.com/evolbioinfo/goalign/blob/master/docs/commands/mask.md
[9] https://github.com/evolbioinfo/goalign/blob/master/docs/commands/subset.md
[10] https://github.com/evolbioinfo/goalign/blob/master/docs/commands/subsites.md
[11] https://github.com/evolbioinfo/goalign/blob/master/docs/commands/compute.md
[12] https://github.com/evolbioinfo/goalign/blob/master/docs/commands/concat.md

```
    -b https://github.com/evolbioinfo/goalign/raw/master/tests/data/test_xz.xz
```

Several alignments are concatenated (from local files or remote servers), *i.e.* sequences from the same taxa are merged into a single sequence. If a sequence is missing in one of the given alignments, it is replaced by gaps.

15) Build bootstrap alignments[13]

```
goalign build seqboot -i https://github.com/evolbioinfo/goalign/raw/master/tests/data/test_xz.xz -n
500 -o boot
```

The input alignment is downloaded from a remote server, and 500 bootstrap replicates are generated locally.

16) Compute bootstrap support[14]

```
gotree compute support fbp -i
https://github.com/evolbioinfo/gotree/raw/master/tests/data/bootstap_inferred_test.nw.gz \
    -b https://github.com/evolbioinfo/gotree/raw/master/tests/data/bootstap_test.nw.gz
```

A reference tree and a set of bootstrap trees are downloaded from a remote server, and bootstrap supports are computed and attached to branches of the reference tree. Both Felsenstein's (FBP) and transfer version (TBE) of the phylogenetic bootstrap are available with: `gotree compute support fbp` and `gotree compute support tbe`).

[13] https://github.com/evolbioinfo/goalign/blob/master/docs/commands/build.md
[14] https://github.com/evolbioinfo/goalign/blob/master/docs/commands/compute.md

Supplementary Figure S1: Representation of the use case workflow and command templates

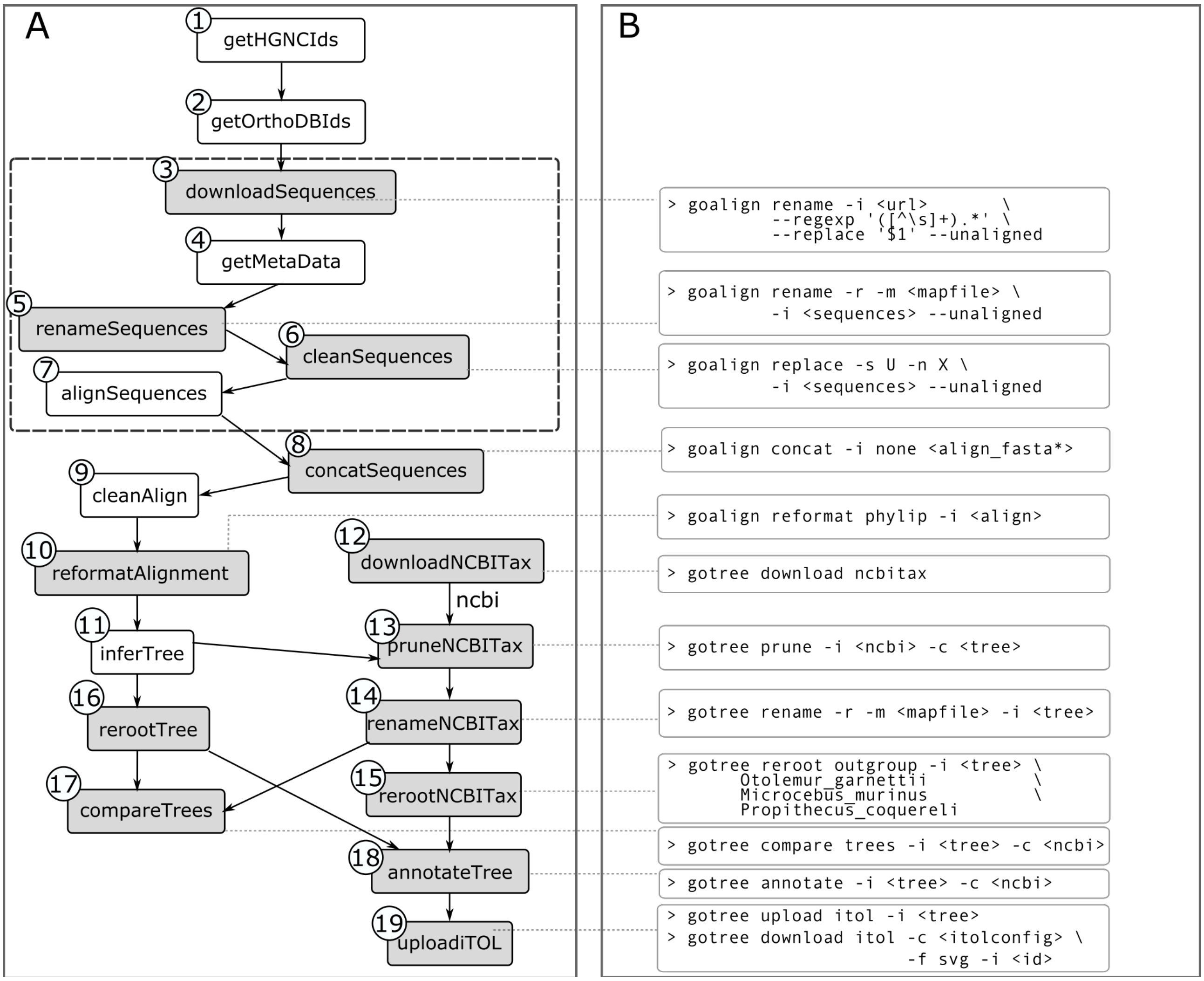

A) This workflow matches RefSeq, HGNC and OrthoDB identifiers from https://doi.org/10.1371/journal.pbio.3000954.s008, and downloads and analyzes 1,315 groups of orthologous proteins having the following characteristics: i) They are shared by at least 90% of the 25 primates (list given below); and ii) only one copy is present in each species (no paralog). The workflow is made of several steps (processes), represented as boxes in the figure. Gray boxes represent steps performed by Gotree/Goalign toolkit. Processes are linked by arrows if results of the upstream process are input of the downstream process. The steps of the workflow, named according to the Nextflow implementation, are the following:

1) **getHGNCIds**: Match RefSeq, NCBI and HGNC (*Eyre et al., NAR 2006*) gene identifiers (dedicated script);
2) **getOrthoDBIds**: Get OrthoDB identifiers of orthologous groups corresponding to HGNC identifiers (orthoDB API);
3) **downloadSequences**: Download sequences of each group and keep only orthoDB ID from the sequence names (Goalign rename command taking the url as input);
4) **getMetaData:** Get species name of each sequence from orthoDB ID (OrthoDB API);
5) **renameSequences**: Rename the sequences using the species names (Goalign);
6) **cleanSequences:** Clean the sequences by removing special characters (Goalign);
7) **alignSequences**: Align the sequences (MAFFT, default options);
8) **concatSequences:** Concatenate all alignments in a single large genomic alignment (Goalign);
9) **cleanAlign:** Clean the alignment (BMGE, with options -t AA -m BLOSUM62 -w 3 -g 0.2 -h 0.5 -b 5);
10) **reformatAlign:** Reformat the alignment into Phylip format (Goalign);
11) **inferTree:** Infer the phylogenetic tree (IQTree, with options -seed 2000 -m MFP -b 100 -nt 20), best model according to Model Finder : JTT+F+R5 (JTT with empirical AA frequencies estimated from the data, and FreeRate model of rates accros sites variability);

12) **DownloadNCBITax:** Download the full NCBI taxonomy in Newick format (Gotree);
13) **pruneNCBITax:** Keep only primate species from NCBI taxonomy (Gotree);
14) **renameNCBITax:** Change species names that differ between orthoDB and NCBI taxonomy (Gotree) ;
15) **rerootNCBITax:** Reroot NCBI taxonomy (Gotree) ;
16) **rerootTree:** Reroot inferred tree using the given outgroup (Gotree);
17) **compareTrees**: Compare both trees in terms of common bi-partitions (Gotree, indicates the number of common and specific bi-partitions);
18) **annotateTree:** Annotate inferred tree with NCBI taxonomy clades (Gotree, indicates for each branch of the tree the closest branch of the NCBI taxonomy in terms of transfer index);
19) **uploadITOL:** Upload the annotated tree to iTOL and store the link to the tree (Gotree).

B) For each step executed using Gotree/Goalign, the template of the command is given in the form "gotree|goalign <command> <subcommand> [options]". Inputs and outputs of most commands / subcommands may be stdin/stdout, allowing for chaining them easily. The first command downloads a set of sequences from OrthoDB and keeps only the OrthoDB identifiers as sequence names; the second command replaces OrthoDB identifiers as sequence names with species names retrieved beforehand.

Supplementary Data 1: Nextflow implementation of the use case

```
params.nboot = 100
params.seed=2000
params.outpath="results"
params.itolconfig= "data/itol_image_config.txt"
params.refseqid = "data/refseq_ids.txt"
params.ncbirenamefile = "data/ncbi_rename.txt"
params.itolkey="none"
params.itolproject="none"

nboot = params.nboot
seed = params.seed
outpath = file(params.outpath)
itolconfig=file(params.itolconfig)
gene2accession=file("ftp://ftp.ncbi.nlm.nih.gov/gene/DATA/gene2accession.gz")
geneinfo=file("ftp://ftp.ncbi.nlm.nih.gov/gene/DATA/gene_info.gz")
refseqid=file(params.refseqid)
ncbirenamefile=file(params.ncbirenamefile)
itolkey=params.itolkey
itolproject=params.itolproject



// Get the NCBI & HGNC ID Related to the RefSeq protein ID given in
//
https://journals.plos.org/plosbiology/article/file?type=supplementary&id=info:doi/10.1371/journal.pbio.3000954.s008&rev=1
process getNCBIIds{
    publishDir "${outpath}", mode: 'copy'

    input:
    file refseqid
    file gene2accession
    file geneinfo

    output:
    file 'refseq_ids_xref.txt'
    file 'refseq_ids_hgnc.txt' into hgncid

    script:
    """
    add_hgnc.pl $refseqid $gene2accession $geneinfo | sort -u >
refseq_ids_xref.txt
    cut -f 3 refseq_ids_xref.txt | sort -u > refseq_ids_hgnc.txt
    """
}



// Get OrthoDB sequences corresponding to NCBI ID
process getOrthoDBIds{
    maxForks 3

    input:
    val hgnc from hgncid.splitText( by: 1 ).map{ v -> v.trim() }

    output:
    file "ids.txt" into protids,protids2

    script:
    """
    curl
'https://v100.orthodb.org/search?query=${hgnc}&ncbi=0&level=9443&species=9443&singlecopy=1&universal=0.9'|jq '.data | join(",")' | sed 's/"//g' | sed
's/,/\\n/g' > ids.txt
    sleep 1
    """
}

protids2.collectFile(name: "all_orthoid.txt").subscribe{file ->
file.copyTo(outpath.resolve(file.name))}

// Download sequences and metadata
process downloadSequences{
    maxForks 1
    tag "${id}"

    input:
    val id from protids.collectFile(name: 'allids.txt').splitText( by: 1 ).map{
v -> v.trim() }.unique().filter{ it.length() > 0 }

    output:
    tuple val(id), file("sequences.fasta") into sequences

    script:
    """
    goalign rename -i "https://v100.orthodb.org/fasta?id=${id}"  --regexp
'([^\\s]+).*' --replace '\$1' --unaligned > sequences.fasta
    sleep 2
    """
}



// Download meta data
process getMapTable{
    maxForks 1
    tag "${id}"

    input:
    set val(id), file(seq) from sequences

    output:
    set val(id), file(seq), file("map.txt") into mapfile
    file "gene.txt" into genefile

    script:
    """
    wget -O align.tab https://v100.orthodb.org/tab?query=${id}
    cut -f 5,6 align.tab > map.txt
    cut -f 1,2 align.tab | tail -n+2 | sort -u > gene.txt
    """
}
```

```
genefile.collectFile(name: 'genes.txt').subscribe{file ->
file.copyTo(outpath.resolve(file.name))}

// Renaming sequences
process renameSequences{
    tag "${id}"

    input:
    set val(id), file(sequences), file(mapfile) from mapfile

    output:
    file "renamed.fasta" into renamed

    script:
    """
    goalign rename -r -m ${mapfile} -i ${sequences} --unaligned | goalign
rename --regexp " " --replace "_" --unaligned  > renamed.fasta
    """
}



// Cleaning sequences
process cleanSequences{
    input:
    file(sequences) from renamed

    output:
    file "cleaned.fasta" into cleaned

    script:
    """
    goalign replace -s U -n X -i ${sequences} -o cleaned.fasta --unaligned
    """
}



// Aligning sequences
process alignSequences{
    input:
    file cleaned

    output:
    file "aligned.fasta" into alignment

    script:
    """
    mafft --quiet ${cleaned} > aligned.fasta
    """
}



// Concatenating all multiple sequence alignments
process concatSequences {
    input:
    file 'align_fasta' from alignment.collect()

    output:
    file "concat.fasta" into concat

    script:
    """
    goalign concat -o concat.fasta -i none align_fasta*
    """
}

// Cleaning the concatenated multiple sequence alignment (BMGE)
process cleanAlign {
    input:
    file align from concat

    output:
    file "cleanalign.fasta" into cleanalign

    script:
    """
    BMGE -i ${align} -t AA -m BLOSUM62 -w 3 -g 0.2 -h 0.5  -b 5 -of
cleanalign.fasta
    """
}



// Reformatting the alignment in Phylip
process reformatAlignment{
    input:
    file cleanalign

    output:
    file "aligned.phylip" into alignmentphylip

    script:
    """
    goalign reformat phylip -i ${cleanalign} -o aligned.phylip
    """
}

// Inferring the species tree from the concatenated
// multiple sequence alignment
process inferTrueTree{
    publishDir "${outpath}", mode: 'copy'

    input:
    file align from alignmentphylip
    val seed

    output:
    file "tree.nw" into tree, tree2

    script:
    """
```

```
    iqtree -s ${align} -seed ${seed} -m MFP -b 100 -nt ${task.cpus}
    mv *.treefile tree.nw
    """
}

// Rerooting the tree
process rerootTree{
    publishDir "${outpath}", mode: 'copy'

    input:
    file tree from tree2

    output:
    file "rerooted.nw" into reroottree1, reroottree2

    script:
    """
    gotree reroot outgroup -i ${tree} -o rerooted.nw Otolemur_garnettii
Microcebus_murinus Propithecus_coquereli
    """
}

// Downloading the NCBI Taxonomy
process downloadNewickTaxonomy {
    output:
    file "ncbi.nw" into ncbitax

    script:
    """
    gotree download ncbitax -o ncbi.nw
    """
}


// Keep the desired species from the NCBI taxonomy
process pruneNCBITax {

    input:
    file tree from tree
    file map from ncbirenamefile
    file ncbi from ncbitax

    output:
    file "ncbi_pruned.nw" into ncbipruned

    script:
    """
    gotree rename -i ${tree} -m ${map} -r -o tmp
    gotree prune -i ${ncbi} -c tmp -o ncbi_pruned.nw
    """
}

// Rename some tips from the NCBI Taxonomy to match
// orthodb species names
process renameNCBITaxonomy {
    input:
    file ncbi from ncbipruned
    file map from ncbirenamefile

    output:
    file "ncbi_rename.nw" into ncbitaxrename

    script:
    """
    gotree rename -i $ncbi -o ncbi_rename.nw -m $map
    """
}

// Reroot the NCBI taxonomy
process rerootNCBITax {

    input:
    file tree from ncbitaxrename

    output:
    file "ncbi_rerooted.nw" into ncbirerooted1, ncbirerooted2

    script:
    """
    gotree reroot outgroup -i ${tree} -o ncbi_rerooted.nw Otolemur_garnettii
Microcebus_murinus Propithecus_coquereli
    """
}


// Annotating the inferred tree
process annotateTree{
    publishDir "${outpath}", mode: 'copy'

    input:
    file tree from reroottree1
    file ncbi from ncbirerooted1

    output:
    file "annotated.nw" into annotated

    script:
    """
    gotree annotate -i ${tree} -c ${ncbi} -o annotated.nw
    """
}


// Comparing inferred tree and NCBI taxonomy branches
process compareTrees{
    publishDir "${outpath}", mode: 'copy'

    input:
    file tree from reroottree2
    file ncbi from ncbirerooted2
```

```
    output:
    file "tree_comparison.txt" into comparison

    script:
    """
    gotree compare trees -i ${tree} -c ${ncbi} > tree_comparison.txt
    """
}

// Uploading tree to ITol &
// Downloading the resulting image
process uploadTree{
    publishDir "${outpath}", mode: 'copy'

    input:
    file tree from annotated
    file itolconfig
    val itolkey
    val itolproject

    output:
    file "tree_url.txt" into iTOLurl
    file "tree_image.svg" into iTOLimage

    script:
    """
    # Upload the tree
    gotree upload itol --name "AnnotatedTree" -i ${tree} --user-id ${itolkey} -
-project ${itolproject} > tree_url.txt
    # We get the iTOL id
    ID=\$(basename \$(cat tree_url.txt ))
    # We Download the image with options defined in data/itol_image_config.txt
    gotree download itol -c ${itolconfig} -f svg -o tree_image.svg -i \$ID
    """
}
```

Supplementary Data 2: List of analyzed primate species (OrthoDB ID: 9443)

- Cercocebus atys
- Chlorocebus sabaeus
- Colobus angolensis palliatus
- Macaca fascicularis
- Macaca mulatta
- Macaca nemestrina
- Mandrillus leucophaeus
- Papio anubis
- Piliocolobus tephrosceles
- Rhinopithecus bieti
- Rhinopithecus roxellana
- Gorilla gorilla gorilla
- Homo sapiens
- Pan paniscus
- Pan troglodytes
- Pongo abelii
- Nomascus leucogenys
- Aotus nancymaae
- Callithrix jacchus
- Carlito syrichta
- Cebus capucinus imitator
- Microcebus murinus
- Otolemur garnettii
- Propithecus coquereli
- Saimiri boliviensis boliviensis

Supplementary Data 3: List of 1,315 orthologous groups from OrthoDB

35432at9443	Solute carrier family 35 member B1
36789at9443	Radial spoke head 1 homolog
27066at9443	POU domain protein
43408at9443	Homeobox C12
32142at9443	Thioredoxin domain containing 15
11455at9443	Microtubule associated protein 6
12380at9443	Centrobin, centriole duplication and spindle assembly protein
30512at9443	Cysteine rich with EGF like domains 2
23782at9443	Serpin family A member 11
29280at9443	Ganglioside induced differentiation associated protein 1
39814at9443	Ras homolog family member J
47676at9443	Fatty acid binding protein 4
27275at9443	dual specificity protein phosphatase 5
16315at9443	Dimethylaniline monooxygenase
21933at9443	INSM transcriptional repressor 2
33961at9443	Acyl-CoA binding domain containing 3
46480at9443	transmembrane protein 252
26393at9443	Arrestin domain containing 2
48705at9443	orexigenic neuropeptide QRFP
13878at9443	selenoprotein O
14869at9443	NudC domain containing 1
35926at9443	Olfactory receptor
13040at9443	conserved oligomeric Golgi complex subunit 2 isoform X1
11387at9443	coiled-coil domain-containing protein 87
22754at9443	Gliomedin
25084at9443	ubiquilin-like protein
48638at9443	Fatty acid binding protein 1
36873at9443	glucagon
36004at9443	Enoyl-CoA hydratase, short chain 1
41871at9443	RNA binding motif protein, X-linked 2
35629at9443	Olfactory receptor
35640at9443	Testis specific serine kinase 3
44093at9443	V-set and transmembrane domain containing 2B
22703at9443	Solute carrier family 19 member 2
40360at9443	Chromosome 3 open reading frame 84
37862at9443	CD40 ligand
27625at9443	zinc finger protein 622
27795at9443	Family with sequence similarity 187 member B
35153at9443	POU domain protein
16111at9443	Phospholipase B-like
27435at9443	otoconin-90
18294at9443	Solute carrier family 32 member 1
27888at9443	taste receptor type 2 member 5
46079at9443	Chromosome 2 open reading frame 50
6770at9443	CTR9 homolog, Paf1/RNA polymerase II complex component
41594at9443	Ras homolog family member D
24154at9443	Ladinin-1
30734at9443	5'-nucleotidase, cytosolic IA
32795at9443	Family with sequence similarity 98 member A
40610at9443	Homeobox C6
28498at9443	Vasopressin V1b receptor
45616at9443	Amphiregulin
23015at9443	Aspartate aminotransferase
47568at9443	glutamyl-tRNA(Gln) amidotransferase subunit C, mitochondrial
39883at9443	olfactory receptor 8B4
12729at9443	DEAQ-box RNA dependent ATPase 1
46698at9443	Chromosome 20 open reading frame 85
23518at9443	Serpin family C member 1
43531at9443	recoverin
47119at9443	Retinol binding protein 2
17118at9443	Kelch like family member 34
26324at9443	cathepsin D
30237at9443	Sodium/potassium-transporting ATPase subunit beta
17598at9443	Zinc finger protein 662
6890at9443	MN1 proto-oncogene, transcriptional regulator
49393at9443	Secreted LY6/PLAUR domain containing 1
22638at9443	Solute carrier family 30 member 1
48422at9443	Peptidyl-tRNA hydrolase domain containing 1
11323at9443	kelch domain-containing protein 7A
38657at9443	taste receptor type 2 member 1
4453at9443	BUB1 mitotic checkpoint serine/threonine kinase
44903at9443	BCL2 related protein A1
16598at9443	DEF6, guanine nucleotide exchange factor
10037at9443	Protein kinase C eta type
19881at9443	Scavenger receptor class B member 2
28254at9443	ETS variant 7
32454at9443	POU domain protein
39253at9443	ferritin, mitochondrial
31663at9443	Phosphatidylinositol 4-kinase type 2 alpha
33818at9443	transmembrane protein 115
42070at9443	Retinol binding protein 1
39125at9443	Transmembrane protein 70
42751at9443	CD68 molecule
22585at9443	Bone morphogenetic protein 7
38043at9443	protein FAM122A
50804at9443	Cyclin-dependent kinases regulatory subunit
37220at9443	Claudin
48796at9443	Chromosome 15 open reading frame 62
43761at9443	Crystallin gamma B
36377at9443	zinc/RING finger protein 4
34691at9443	Gastrulation brain homeobox 2
19314at9443	inositol 1,4,5-trisphosphate receptor-interacting protein-like 2
28692at9443	Dynein cytoplasmic 2 light intermediate chain 1
39964at9443	StAR related lipid transfer domain containing 5
20753at9443	PLAG1 like zinc finger 1
33252at9443	melanocortin receptor 4
28453at9443	Chromosome 2 open reading frame 69
46047at9443	Myosin light chain 1
32564at9443	UbiA prenyltransferase domain containing 1
39038at9443	Yip1 interacting factor homolog A, membrane trafficking protein
17438at9443	Cholinergic receptor nicotinic delta subunit
49824at9443	LOW QUALITY PROTEIN: BH3-like motif-containing cell death inducer
35448at9443	TNF alpha induced protein 6
42368at9443	ubiquitin-conjugating enzyme E2 G1
38397at9443	Adrenoceptor beta 3
28601at9443	Transforming growth factor beta regulator 1
44885at9443	Chromosome 8 open reading frame 46
5241at9443	Kinesin family member 11
7082at9443	tubulin monoglycylase TTLL3 isoform X1
37714at9443	Homeobox A10
15529at9443	Ribosomal RNA processing 1B
43452at9443	Protein lin-7 homolog
30848at9443	Tartrate-resistant acid phosphatase type 5
41251at9443	Claudin
14582at9443	Biotinidase
23630at9443	forkhead box protein O4
40694at9443	alpha-ketoglutarate-dependent dioxygenase alkB homolog 6 isoform X1
52140at9443	adipogenesis regulatory factor
17416at9443	ATPase H+ transporting V1 subunit B1
39630at9443	Single-pass membrane protein with coiled-coil domains 1
38448at9443	Fc fragment of IgE receptor Ia
37877at9443	Enoyl-CoA delta isomerase 1
47021at9443	Retinol binding protein 5
27421at9443	NADH dehydrogenase
18221at9443	Translocase of outer mitochondrial membrane 70
26245at9443	transmembrane protease serine 11D
34770at9443	RCSD domain containing 1
48816at9443	Succinate dehydrogenase complex assembly factor 3
26703at9443	Synaptotagmin 13
45603at9443	DNL-type zinc finger protein
50242at9443	splicing factor 3B subunit 5
31027at9443	Secreted frizzled related protein 4
37045at9443	Junctional adhesion molecule 2
40827at9443	B9 domain containing 1
30646at9443	Inositol monophosphatase domain containing 1
40001at9443	YjeF N-terminal domain containing 3
21665at9443	lymphocyte cytosolic protein 2
19837at9443	dystrotelin
34299at9443	G protein-coupled receptor 150
37113at9443	N(alpha)-acetyltransferase 30, NatC catalytic subunit
40661at9443	SS18L1, nBAF chromatin remodeling complex subunit
31118at9443	MOS proto-oncogene, serine/threonine kinase
29443at9443	Dol-P-Man
20543at9443	Matrilin 3
14740at9443	Olfactomedin like 2A
39311at9443	Transmembrane protein 65
43017at9443	Ubiquitin conjugating enzyme E2 S
10232at9443	Phosphoinositide-3-kinase adaptor protein 1
35841at9443	Calbindin 2
26486at9443	Zinc finger protein 764
51031at9443	Chromosome 1 open reading frame 189
28956at9443	Ankyrin repeat and sterile alpha motif domain containing 4B
47181at9443	Cornifelin
28323at9443	Abhydrolase domain containing 1
33770at9443	Potassium channel tetramerization domain containing 13
43580at9443	NK6 homeobox 1
27827at9443	Cathepsin H
27859at9443	Major facilitator superfamily domain containing 9
15419at9443	Dolichyl-diphosphooligosaccharide--protein glycosyltransferase subunit 1
37892at9443	keratin-associated protein 24-1
34633at9443	Solute carrier family 35 member G1
26731at9443	ameloblastin
45335at9443	Chromosome X open reading frame 66
39541at9443	Fat storage inducing transmembrane protein 1
8928at9443	NOP14 nucleolar protein
13279at9443	Adducin 3
46086at9443	Endothelin 2
34944at9443	RING finger protein 148
33502at9443	Neurogenic differentiation factor
640at9443	ATP-binding cassette sub-family A member 2
34488at9443	Dermokine
28848at9443	nucleosome assembly protein 1-like 2
41673at9443	RAB39B, member RAS oncogene family
22284at9443	Proteasome 26S subunit, non-ATPase 3
32631at9443	secreted frizzled-related protein 2
37429at9443	zinc finger CCCH-type antiviral protein 1-like
28764at9443	Chromosome 1 open reading frame 87
28700at9443	Dual specificity protein phosphatase
43545at9443	Paired like homeobox 2a
46432at9443	Ubiquitin conjugating enzyme E2 L6
6836at9443	mutS protein homolog 4
28958at9443	Transmembrane p24 trafficking protein family member 8
26544at9443	Tudor domain containing 10
25495at9443	AlkB homolog 1, histone H2A dioxygenase
29624at9443	Replication factor C subunit 5
30164at9443	Mesoderm posterior bHLH transcription factor 2
47093at9443	Ubiquitously expressed prefoldin like chaperone
40027at9443	Transmembrane protein 147
16995at9443	thrombomodulin
19145at9443	interferon regulatory factor 2-binding protein 1
24704at9443	Nerve growth factor receptor
7006at9443	Transcriptional regulating factor 1
12531at9443	exocyst complex component 8
796at9443	Human immunodeficiency virus type I enhancer binding protein 2
38840at9443	Kruppel like factor 9
39801at9443	Chromosome 16 open reading frame 78
11160at9443	Transporter 1, ATP binding cassette subfamily B member
50543at9443	DnaJ heat shock protein family (Hsp40) member C15
40757at9443	sepiapterin reductase
29949at9443	Solute carrier family 30 member 8
15117at9443	WD repeat domain 43
21072at9443	Solute carrier family 16 member 2
46433at9443	IQ motif containing F2
38975at9443	Secretory carrier-associated membrane protein
34362at9443	Mitochondrial ribosomal protein L2
38601at9443	2-aminoethanethiol dioxygenase
25161at9443	wnt inhibitory factor 1
21618at9443	Lecithin-cholesterol acyltransferase
6127at9443	Poly
39459at9443	Stanniocalcin 1
15357at9443	Phosphodiesterase 12
19162at9443	Ectonucleoside triphosphate diphosphohydrolase 8
36578at9443	CutC copper transporter
28847at9443	Methyltransferase like 14
46500at9443	transmembrane protein 160
30743at9443	Family with sequence similarity 221 member B
42381at9443	ciliary neurotrophic factor
17408at9443	HERV-H LTR-associating protein 1
48918at9443	splicing factor 3B subunit 6
33083at9443	Olfactory receptor
36066at9443	V-set and immunoglobulin domain containing 2
41339at9443	RAB25, member RAS oncogene family
12429at9443	Coiled-coil domain containing 142
45948at9443	Claudin

3774at9443	retinol-binding protein 3	43656at9443	Claudin
23820at9443	LanC like 3	45641at9443	Chromosome 11 open reading frame 88
21424at9443	WD repeat domain 88	22773at9443	Ectonucleoside triphosphate diphosphohydrolase 2
45285at9443	PYD and CARD domain containing	49354at9443	prefoldin subunit 2
24692at9443	Adrenoceptor beta 2	49128at9443	Chromosome 11 open reading frame 97
38372at9443	E3 ubiquitin-protein ligase	51627at9443	uncharacterized protein C11orf71 homolog
13333at9443	SPOC domain containing 1	36548at9443	matrilysin
42824at9443	DAN domain family member 5	48928at9443	Thiosulfate sulfurtransferase like domain containing 1
19053at9443	Tyrosine hydroxylase	10158at9443	Unkempt family zinc finger
3739at9443	golgi glycoprotein 1	11004at9443	cadherin-15
21862at9443	Lipase	46332at9443	musculin
40688at9443	TP53 regulating kinase	26001at9443	cytoskeleton-associated protein 4
8148at9443	Spalt like transcription factor 2	44207at9443	Protein yippee-like
42144at9443	Stromal cell derived factor 2 like 1	35441at9443	ceroid-lipofuscinosis neuronal protein 6
12721at9443	Tubulin tyrosine ligase like 12	21383at9443	interleukin-1 receptor-associated kinase 1
37685at9443	three prime repair exonuclease 2	40715at9443	Receptor expression-enhancing protein
5126at9443	Sodium/potassium-transporting ATPase subunit alpha	39236at9443	Zinc finger protein 414
31695at9443	Calcium homeostasis modulator family member 4	30198at9443	Sulfotransferase
32227at9443	melanocortin receptor 5	31817at9443	Pim-2 proto-oncogene, serine/threonine kinase
33178at9443	Somatomedin B and thrombospondin type 1 domain containing	26160at9443	beta-1,4-galactosyltransferase 5
29720at9443	2-oxoglutarate and iron dependent oxygenase domain containing 2	44887at9443	Proteasome subunit beta type
39406at9443	NK2 homeobox 6	48472at9443	Oligodendrocytic myelin paranodal and inner loop protein
48647at9443	phospholipase A2	50143at9443	C-C motif chemokine 22
46678at9443	BTG anti-proliferation factor 2	36234at9443	Transmembrane protein 150A
28831at9443	Cytotoxic and regulatory T cell molecule	29089at9443	Signal regulatory protein beta 2
37098at9443	Distal-less homeobox 3	46543at9443	serine/arginine-rich splicing factor 1
43816at9443	Mitochondrial ribosomal protein S23	24851at9443	Indoleamine 2,3-dioxygenase 2
24320at9443	uncharacterized protein C3orf30 homolog	9956at9443	ATP binding cassette subfamily D member 2
47007at9443	Centrin 3	23340at9443	Coiled-coil domain containing 17
40278at9443	INO80 complex subunit B	22819at9443	reticulon-4 receptor
34596at9443	Methylthioribose-1-phosphate isomerase	10211at9443	ATP binding cassette subfamily B member 7
27237at9443	actin-like protein 9	40195at9443	E3 ubiquitin-protein ligase RNF187
35895at9443	proline synthase co-transcribed bacterial homolog protein	41953at9443	centromere protein H
49672at9443	EP300-interacting inhibitor of differentiation 2B	48116at9443	Coiled-coil domain containing 182
38613at9443	Shisa family member 2	25777at9443	Phospholipid phosphatase related 2
24600at9443	Receptor interacting serine/threonine kinase 3	44873at9443	Vacuolar protein sorting 25 homolog
32125at9443	F-actin-capping protein subunit alpha-3	29945at9443	Translocating chain-associated membrane protein
44287at9443	Lens fiber membrane intrinsic protein	35117at9443	Homeobox B13
38475at9443	Glutaredoxin and cysteine rich domain containing 2	42213at9443	Sugar transporter SWEET
30588at9443	Opsin 1, short wave sensitive	44067at9443	Adenine phosphoribosyltransferase
32731at9443	Aquaporin 3 (Gill blood group)	17243at9443	Arylsulfatase B
15097at9443	Tripartite motif containing 23	47700at9443	refilin B
14840at9443	CD93 molecule	32073at9443	ATPase H+/K+ transporting beta subunit
44435at9443	B-cell lymphoma/leukemia 10	32860at9443	Metaxin 3
32852at9443	CRK like proto-oncogene, adaptor protein	21943at9443	Hemopexin
22012at9443	Angiopoietin like 3	20148at9443	Leucine rich repeat containing 47
15809at9443	inactive serine/threonine-protein kinase PLK5	30676at9443	Galectin
19664at9443	N-lysine methyltransferase SETD6	46599at9443	Troponin C1, slow skeletal and cardiac type
12449at9443	retrotransposon-derived protein PEG10 isoform 1	23857at9443	Solute carrier family 37 member 4
27907at9443	Annexin	45080at9443	Interleukin-18
38203at9443	Vacuolar-sorting protein SNF8	34763at9443	Ankyrin repeat domain 63
47824at9443	mitochondrial import inner membrane translocase subunit Tim22	49061at9443	H1 histone family member X
40116at9443	transcription factor MafB	45657at9443	Ribosomal RNA processing 36
50388at9443	Interleukin-2	36344at9443	Phosphomannomutase
13044at9443	Solute carrier family 44 member 3	42282at9443	Cilia and flagella associated protein 20
12121at9443	Solute carrier family 39 member 12	27286at9443	IQ motif containing D
21077at9443	NADPH oxidase activator 1 isoform X1	30702at9443	Hyaluronan and proteoglycan link protein 4
32549at9443	Ankyrin repeat domain 1	46422at9443	DnaJ heat shock protein family (Hsp40) member C24
20322at9443	Aconitate decarboxylase 1	43745at9443	Pentaxin
35257at9443	TruB pseudouridine synthase family member 1	11431at9443	TLR4 interactor with leucine rich repeats
15669at9443	tetratricopeptide repeat protein 24	50851at9443	Glutamate rich 4
25395at9443	Mitogen-activated protein kinase	9618at9443	Aldehyde dehydrogenase family 16 member A1
33144at9443	CTD small phosphatase like	38117at9443	Phosphoinositide-3-kinase interacting protein 1
34473at9443	synapse-associated protein 1	50314at9443	guanylate cyclase activator 2B
38717at9443	CD79a molecule	38378at9443	Thioredoxin related transmembrane protein 1
38350at9443	Chymase 1	8195at9443	AP-2 complex subunit alpha
35415at9443	Tumor necrosis factor ligand superfamily member	39390at9443	tumor necrosis factor ligand superfamily member 15
36068at9443	T cell leukemia homeobox 3	20158at9443	BEN domain containing 2
25396at9443	GDNF family receptor alpha like	31390at9443	Free fatty acid receptor 4
37005at9443	SLAM family member 7	29571at9443	Testis specific serine kinase 4
44713at9443	uncharacterized protein C6orf47 homolog	50738at9443	Serine peptidase inhibitor, Kazal type 7 (putative)
25214at9443	Microspherule protein 1	32604at9443	eukaryotic translation initiation factor 3 subunit G
18818at9443	Matrix metalloproteinase	46200at9443	Intercellular adhesion molecule 4 (Landsteiner-Wiener blood group)
42464at9443	Transcription factor SOX	13733at9443	Zinc finger protein 599
14516at9443	Growth factor receptor bound protein 10	24504at9443	CDC like kinase 2
39079at9443	Interleukin 22 receptor subunit alpha 2	39562at9443	retinoschisin
31002at9443	UTP3, small subunit processome component	43935at9443	Glutathione peroxidase
18514at9443	Complement C9	42305at9443	transmembrane emp24 domain-containing protein 7
24030at9443	Multiple EGF like domains 9	28018at9443	Mitochondrial ribosomal protein L37
20604at9443	NMDA receptor synaptonuclear signaling and neuronal migration factor isoform X1	24325at9443	carboxypeptidase B
30887at9443	Glycerophosphodiester phosphodiesterase domain containing 3	26427at9443	Leiomodin 2
38134at9443	Calcium homeostasis modulator family member 6	28126at9443	Mex-3 RNA binding family member A
18860at9443	pre-mRNA-processing factor 19	25044at9443	kaptin
12030at9443	Lipase maturation factor	47970at9443	Transmembrane protein 207
34348at9443	Homeobox A2	14157at9443	Zinc finger SWIM-type containing 2
42686at9443	spermatid maturation protein 1	41669at9443	regulator of G-protein signaling 1
13287at9443	sulfhydryl oxidase 2	29328at9443	Protein Wnt
27810at9443	Spectrin repeat containing nuclear envelope family member 4	31535at9443	G-protein coupled receptor 20
20859at9443	cartilage matrix protein	13317at9443	Interleukin 27 receptor subunit alpha
28751at9443	NIPA like domain containing 4	27781at9443	CD5 molecule like
43794at9443	Clarin 2	44083at9443	Sodium voltage-gated channel beta subunit 2
25792at9443	Nectin cell adhesion molecule 2	40137at9443	Exosome component 5
32402at9443	peroxisome biogenesis factor 13	6489at9443	Espin like
28837at9443	homeobox protein aristaless-like 4	51324at9443	neuropeptide S
50261at9443	LSM4 homolog, U6 small nuclear RNA and mRNA degradation associated	725at9443	lipoxygenase homology domain-containing protein 1 isoform X1
22842at9443	Cytidine monophosphate N-acetylneuraminic acid synthetase	27737at9443	Growth differentiation factor 2
39073at9443	Hexamethylene bisacetamide inducible 1	22097at9443	alpha-(1,3)-fucosyltransferase 4
49344at9443	Interferon induced transmembrane protein 5	18299at9443	Gap junction protein
36727at9443	TP53 induced glycolysis regulatory phosphatase	44584at9443	Tetratricopeptide repeat domain 9
25528at9443	protein FAM81B	47964at9443	cystatin-like 1
20415at9443	Kremen protein	51565at9443	S100 calcium binding protein A12
35468at9443	zinc finger DHHC-type containing 19	49502at9443	zinc finger protein 593
36806at9443	Leucine rich repeat containing 58	41858at9443	Folliculogenesis specific bHLH transcription factor
46139at9443	Cytidine deaminase	20279at9443	Adenylosuccinate synthetase isozyme 2
35986at9443	ubiquitin-conjugating enzyme E2 Z	40220at9443	Homeobox A6
34382at9443	Solute carrier family 51 alpha subunit	41211at9443	Protease associated domain containing 1
21108at9443	matrix metallopeptidase 8	40300at9443	Quinoid dihydropteridine reductase
35746at9443	BCDIN3 domain containing RNA methyltransferase	34270at9443	taste receptor type 2 member 39
25730at9443	Choline kinase beta	19333at9443	Testis expressed metallothionein like protein
8722at9443	Amine oxidase	8518at9443	AE binding protein 1
28772at9443	TBC1 domain family member 21	33107at9443	Na(+)/H(+) exchange regulatory cofactor NHE-RF1
32742at9443	Dehydrogenase/reductase 7C	30485at9443	Arginase
30565at9443	thymidylate synthase	26877at9443	pre-mRNA-splicing factor RBM22
42606at9443	Non-specific cytotoxic cell receptor protein 1 homolog (zebrafish)	28604at9443	Neuromedin U receptor 1

37295at9443	Solute carrier family 35 member E4
42439at9443	Amelotin
46194at9443	charged multivesicular body protein 4c
33417at9443	Stomatin like 2
35347at9443	Serine protease 57
27975at9443	Keratin 20
22404at9443	Lactate dehydrogenase D
30562at9443	nucleosome assembly protein 1-like 3
51098at9443	Liver enriched antimicrobial peptide 2
16490at9443	EGF like domain multiple 6
34933at9443	STIP1 homology and U-box containing protein 1
6824at9443	DExD/H-box helicase 58
33169at9443	BarH like homeobox 2
963at9443	Voltage-dependent N-type calcium channel subunit alpha
48373at9443	BCL tumor suppressor 7A
33800at9443	olfactory receptor 13J1
50950at9443	Transmembrane protein 35B
31728at9443	nucleoside diphosphate-linked moiety X motif 19
9893at9443	DEAD-box helicase 1
46447at9443	charged multivesicular body protein 1b
43195at9443	C-type lectin domain family 4 member E
25682at9443	Ankyrin repeat and SOCS box containing 16
42555at9443	Fibroblast growth factor
16439at9443	Major facilitator superfamily domain containing 6 like
31488at9443	Solute carrier family 10 member 4
31994at9443	Solute carrier family 35 member E3
32897at9443	Beta-1,3-N-acetylglucosaminyltransferase
36494at9443	coiled-coil domain containing 28B
34801at9443	Olfactory receptor
27120at9443	ST8 alpha-N-acetyl-neuraminide alpha-2,8-sialyltransferase 3
25362at9443	transcription factor IIIA
17048at9443	TBC1 domain family member 24 isoform X1
23443at9443	sphingosine-1-phosphate phosphatase 1
42810at9443	ADP ribosylation factor like GTPase 14
50691at9443	basic leucine zipper transcriptional factor ATF-like
41130at9443	Fanconi anemia core complex associated protein 24
37010at9443	Atonal bHLH transcription factor 1
33452at9443	melanocortin receptor 3
47455at9443	Profilin
33913at9443	Ankyrin repeat domain 23
27619at9443	G protein-coupled receptor 151
25333at9443	One cut domain family member
14362at9443	Complement C8 beta chain
41732at9443	Myelin protein zero like 3
21445at9443	peptidyl-prolyl cis-trans isomerase FKBP5
46614at9443	leucine rich adaptor protein 1-like
29906at9443	Dual specificity protein phosphatase
13303at9443	Crooked neck pre-mRNA splicing factor 1
17868at9443	uncharacterized protein C2orf81 homolog isoform X1
40824at9443	Retina and anterior neural fold homeobox
39909at9443	Ribosome assembly factor mrt4
38910at9443	Homeobox protein
6063at9443	VPS50, EARP/GARPII complex subunit
39639at9443	tumor necrosis factor ligand superfamily member 6
37547at9443	Tubulin epsilon and delta complex 1
32910at9443	VPS26 endosomal protein sorting factor C
44084at9443	MAGE family member H1
43559at9443	Ankyrin repeat domain 22
39123at9443	Small nuclear ribonucleoprotein polypeptide A'
42829at9443	Transmembrane 4 L six family member 20
27926at9443	olfactory receptor 1I1
18378at9443	T-box 21
43036at9443	transmembrane protein 179
25122at9443	early growth response protein 1
18877at9443	T-complex protein 1 subunit delta
17982at9443	Aldehyde dehydrogenase 9 family member A1
23077at9443	PWP1 homolog, endonuclein
42108at9443	Ras homolog family member B
40895at9443	WW domain binding protein 1
45417at9443	Palmitoyltransferase
46801at9443	SCP2 sterol-binding domain-containing protein 1
30001at9443	Isthmin 2
34746at9443	taste receptor type 2 member 3
38761at9443	insulin-like growth factor-binding protein 1
28815at9443	Angiotensin II receptor type 2
46592at9443	Calmodulin like 3
28438at9443	Glucose-6-phosphatase
42421at9443	MAGE family member F1
51738at9443	Chromosome 10 open reading frame 99
28135at9443	Nodal growth differentiation factor
2666at9443	Synemin
37831at9443	Acrosomal vesicle protein 1
2988at9443	ATP-dependent RNA helicase DHX29
44834at9443	Immediate early response 5
47668at9443	Charged multivesicular body protein 6
30038at9443	Stromal cell derived factor 4
25457at9443	Cathepsin E
27862at9443	Golgi reassembly stacking protein 1
30950at9443	cAMP responsive element binding protein 3
50806at9443	Coiled-coil-helix-coiled-coil-helix domain containing 7
44769at9443	Methionine sulfoxide reductase B2
43029at9443	RAB12, member RAS oncogene family
18462at9443	Lengsin, lens protein with glutamine synthetase domain
45390at9443	Centrin 1
39986at9443	Membrane spanning 4-domains A15
40550at9443	UBX domain protein 8
43326at9443	High mobility group box 4
23685at9443	Mitogen-activated protein kinase kinase 7
43427at9443	Neuromedin S
5280at9443	Sodium/potassium-transporting ATPase subunit alpha
45120at9443	Killer cell lectin like receptor G1
21806at9443	Fibrinogen gamma chain
24347at9443	Reticulon 4 interacting protein 1
45529at9443	homeobox protein SEBOX
36403at9443	translational activator of cytochrome c oxidase 1
31508at9443	olfactory receptor 52B2
50338at9443	Keratin associated protein 7-1 (gene/pseudogene)
17073at9443	interferon regulatory factor 2-binding protein-like
38648at9443	Potassium channel regulator
43434at9443	ADP ribosylation factor like GTPase 4D
22535at9443	Family with sequence similarity 217 member A
32769at9443	diacylglycerol O-acyltransferase 2-like protein 6
38962at9443	Interferon induced protein 35
43131at9443	lymphocyte antigen 6 complex locus protein G5b
25441at9443	Solute carrier family 46 member 1
49259at9443	testis-specific H1 histone
14971at9443	Delta like canonical Notch ligand 3
45472at9443	CD70 antigen
28868at9443	GRINL1A complex locus 1
46409at9443	NK3 homeobox 1
43634at9443	Mitochondrial ribosomal protein L13
49864at9443	platelet basic protein
34768at9443	Zinc finger CCHC-type containing 3
15766at9443	conserved oligomeric Golgi complex subunit 8
31852at9443	RNA polymerase III subunit D
24373at9443	Pleckstrin homology domain containing O2
33658at9443	Relaxin/insulin like family peptide receptor 4
49199at9443	Protein phosphatase 1 regulatory inhibitor subunit 14A
50384at9443	LOW QUALITY PROTEIN: prostate and breast cancer overexpressed gene 1 protein
33143at9443	taste receptor type 2 member 40
20784at9443	Zinc finger protein 641
28344at9443	Kelch domain containing 9
49091at9443	Neuromedin U
38639at9443	Homeobox protein
20937at9443	Solute carrier family 2 member 2
32236at9443	Starch binding domain 1
18944at9443	Angiopoietin 1
17063at9443	Beta-hexosaminidase
33356at9443	olfactory receptor 51A7
42547at9443	Chromosome 18 open reading frame 21
10681at9443	MYB proto-oncogene, transcription factor
8373at9443	Complement C7
11346at9443	tubulin polyglutamylase TTLL11
24710at9443	StAR related lipid transfer domain containing 3
46560at9443	keratin-associated protein 11-1
7237at9443	Kinesin family member 17
40104at9443	NECAP endocytosis associated 1
22644at9443	Aldehyde dehydrogenase 5 family member A1
23104at9443	Ankyrin repeat and SOCS box containing 10
22303at9443	sedoheptulokinase
24487at9443	bone morphogenetic protein 10
20120at9443	RAN binding protein 3 like
29432at9443	Zinc activated ion channel
44659at9443	interferon beta
30760at9443	Phospholipid phosphatase related 5
50934at9443	TP53-regulated inhibitor of apoptosis 1
50042at9443	mediator of RNA polymerase II transcription subunit 9
38750at9443	2-oxoglutarate and iron dependent oxygenase domain containing 3
47146at9443	Chromosome 9 open reading frame 116
21890at9443	Cytochrome P450 family 27 subfamily B member 1
29946at9443	Exo/endonuclease G
49499at9443	Chromosome 11 open reading frame 86
51498at9443	potassium voltage-gated channel subfamily E regulatory beta subunit 5
35601at9443	Aquaporin 5
40867at9443	Phosphatidylcholine transfer protein
46769at9443	Mitochondrial ribosomal protein L17
6508at9443	Smith-Magenis syndrome chromosome region, candidate 8
19484at9443	Coiled-coil domain containing 183
31766at9443	Decapping enzyme, scavenger
28320at9443	Cyclin J like
25658at9443	Sad1 and UNC84 domain containing 5
24432at9443	Vasopressin V1a receptor
40182at9443	Leucine rich repeat containing 10B
16693at9443	Protein phosphatase 1 regulatory subunit 3F
44513at9443	protein TNT
40013at9443	protein MAK16 homolog
37783at9443	Chromosome 5 open reading frame 51
28351at9443	Transmembrane protein 171
37030at9443	Dolichyl-phosphate mannosyltransferase subunit 1, catalytic
12859at9443	Component of oligomeric golgi complex 6
44284at9443	Neuronal calcium sensor 1
43377at9443	Ferredoxin 2
38337at9443	Proline rich 22
49292at9443	Leucine rich single-pass membrane protein 2
12196at9443	Electron transfer flavoprotein dehydrogenase
45489at9443	Paired like homeobox 2b
25111at9443	disrupted in renal carcinoma protein 2
25116at9443	nuclear fragile X mental retardation-interacting protein 1
46150at9443	Tetratricopeptide repeat domain 32
23522at9443	carboxypeptidase E
35703at9443	Isochorismatase domain containing 1
40567at9443	RAB11B, member RAS oncogene family
30192at9443	PNMA family member 1
30907at9443	Annexin
49072at9443	Mediator of RNA polymerase II transcription subunit 11
9915at9443	N-ethylmaleimide sensitive factor, vesicle fusing ATPase
27725at9443	phosphorylated adapter RNA export protein
26687at9443	Glucose-6-phosphatase
40236at9443	POU class 2 associating factor 1
38218at9443	Lymphatic vessel endothelial hyaluronan receptor 1
19204at9443	nuclear receptor subfamily 1 group D member 1
16430at9443	REL proto-oncogene, NF-kB subunit
19714at9443	Coiled-coil domain containing 85A
37129at9443	Ubiquitin thioesterase
37505at9443	Integral membrane protein 2A
50129at9443	GADD45G interacting protein 1
43782at9443	5-formyltetrahydrofolate cyclo-ligase
28213at9443	Inhibin beta E subunit
44218at9443	Sodium voltage-gated channel beta subunit 4
45874at9443	Titin-cap
15122at9443	striatin-4 isoform X1
43097at9443	voltage-dependent calcium channel gamma-1 subunit
15957at9443	N-acetylglucosamine-6-sulfatase
13338at9443	rho GTPase-activating protein 40
41468at9443	Myomaker, myoblast fusion factor
19261at9443	Zinc finger protein 219
892at9443	Polycystin family receptor for egg jelly
50274at9443	Dynein light chain roadblock
48702at9443	Gametocyte specific factor 1 like
39362at9443	Hypoxanthine phosphoribosyltransferase
14218at9443	NDC80, kinetochore complex component
45112at9443	Achaete-scute family bHLH transcription factor 3
23002at9443	keratin-associated protein 16-1
43960at9443	Pre-mRNA processing factor 38B
26847at9443	Solute carrier family 46 member 2
38759at9443	Family with sequence similarity 3 member B
31713at9443	keratin-associated protein 29-1
2993at9443	ArfGAP with RhoGAP domain, ankyrin repeat and PH domain 1

38041at9443	olfactory receptor 10K2
44543at9443	Angiopoietin like 8
1784at9443	DNA-directed RNA polymerase II subunit RPB1
22848at9443	Phosphoserine aminotransferase
43387at9443	Sulfhydryl oxidase
35878at9443	Proteasome subunit beta type
26689at9443	SRY-box 7
48388at9443	transmembrane protein 170B
807at9443	Voltage-dependent P/Q-type calcium channel subunit alpha
20166at9443	NADH dehydrogenase
47911at9443	otoraplin
44836at9443	Chromosome 17 open reading frame 98
41252at9443	GrpE protein homolog
16695at9443	Family with sequence similarity 83 member C
22358at9443	Adaptor related protein complex 2 mu 1 subunit
25065at9443	fibrous sheath CABYR-binding protein
7578at9443	General transcription factor IIIC subunit 3
22612at9443	zinc finger protein 597
42227at9443	CD320 antigen isoform X1
33092at9443	olfactory receptor 51S1
9380at9443	threonine synthase-like 1
30508at9443	Potassium two pore domain channel subfamily K member 18
48063at9443	Caveolae associated protein 3
22001at9443	Cilia and flagella associated protein 53
25562at9443	N-acetylglucosamine-6-phosphate deacetylase
10896at9443	Zinc finger SWIM-type containing 3
16310at9443	Werner helicase interacting protein 1
32984at9443	Visual system homeobox 2
49736at9443	histone H1t
45761at9443	translocator protein
28608at9443	Sperm associated antigen 8
42768at9443	Biliverdin reductase B
30349at9443	POU domain, class 3, transcription factor 2
16319at9443	RNA pseudouridylate synthase domain containing 2
42828at9443	Guanylate cyclase activator 1C
49861at9443	Chromosome 20 open reading frame 202
29975at9443	G protein-coupled receptor 139
25967at9443	bone morphogenetic protein 15
30771at9443	Homeobox C10
45405at9443	zinc finger matrin-type protein 2
35982at9443	TWIST neighbor
25923at9443	cornulin
42901at9443	Phosphomevalonate kinase
50208at9443	CART prepropeptide
26831at9443	Olfactomedin like 3
36934at9443	Enoyl-CoA hydratase domain containing 2
48609at9443	Chromosome 16 open reading frame 91
31194at9443	retinol dehydrogenase 10
46179at9443	Signaling threshold regulating transmembrane adaptor 1
22751at9443	chitobiosyldiphosphodolichol beta-mannosyltransferase
39869at9443	L-xylulose reductase
30420at9443	SH3 and cysteine rich domain 2
48041at9443	GON7, KEOPS complex subunit
47116at9443	Proprotein convertase subtilisin/kexin type 1 inhibitor
14601at9443	MAGE family member L2
37905at9443	Insulin like growth factor binding protein 5
48714at9443	mitochondrial fission 1 protein
31960at9443	Calcium homeostasis modulator family member 5
13534at9443	Transporter
17393at9443	Basal cell adhesion molecule (Lutheran blood group)
27222at9443	growth/differentiation factor 8
24608at9443	Protein O-glucosyltransferase 1
25961at9443	Transmembrane protein with EGF like and two follistatin like domains 2
43139at9443	Tetraspanin 13
44673at9443	Transmembrane protein 52
27637at9443	Protein phosphatase, Mg2+/Mn2+ dependent 1L
41608at9443	tumor necrosis factor ligand superfamily member 9
38514at9443	Gamma-glutamylcyclotransferase
49280at9443	Prostate and testis expressed 1
42585at9443	Cyclin dependent kinase 2 interacting protein
36614at9443	Non imprinted in Prader-Willi/Angelman syndrome 1
22769at9443	Lipopolysaccharide binding protein
31260at9443	Caveolae associated protein 2
22833at9443	AT-rich interaction domain 3B
49759at9443	Migration and invasion enhancer 1
5950at9443	Aminopeptidase
45924at9443	BCL2 associated agonist of cell death
19848at9443	Calcitonin receptor
48430at9443	Serglycin
9983at9443	SPG7, paraplegin matrix AAA peptidase subunit
46820at9443	Achaete-scute family bHLH transcription factor 5
21881at9443	tRNA methyltransferase 61B
42152at9443	glutathione peroxidase 2
33482at9443	Solute carrier family 25 member 20
32575at9443	Sorting nexin family member 21
25454at9443	Protein Wnt
10078at9443	KIAA1522
49005at9443	protein ripply3
17345at9443	Breast cancer type 1 susceptibility protein homolog
26907at9443	Alcohol dehydrogenase 4 (class II), pi polypeptide
42503at9443	Distal-less homeobox 4
32026at9443	junctional adhesion molecule 3
32835at9443	Homeobox A5
21158at9443	dolichol kinase
29356at9443	Zona pellucida binding protein 2
41108at9443	UTP23, small subunit processome component
30799at9443	Solute carrier family 25 member 32
36086at9443	interleukin-1 beta
33409at9443	hydroxycarboxylic acid receptor 1
23249at9443	transmembrane protein 151A
31223at9443	Somatostatin receptor 4
4838at9443	Nucleolar protein 6
44468at9443	Fibroblast growth factor
31393at9443	Pentraxin 3
29684at9443	Glutaminyl-peptide cyclotransferase
34438at9443	Cilia and flagella associated protein 77
23603at9443	Fc receptor like 2
23569at9443	DNA primase small subunit
28628at9443	Fructose-bisphosphate aldolase
40639at9443	Dual specificity phosphatase 19
39957at9443	granzyme K
39832at9443	caspase-14
39554at9443	AMMECR1
38621at9443	out at first protein homolog
6572at9443	GRB2 associated regulator of MAPK1 subtype 1
44489at9443	Thioredoxin domain containing 12
43137at9443	Vitelline membrane outer layer 1 homolog
50986at9443	orexin
29825at9443	WD repeat domain 54
25811at9443	Neuronal pentraxin 1
29620at9443	Protein phosphatase 1 regulatory subunit 15A
42549at9443	noggin
39896at9443	Telomere repeat binding bouquet formation protein 2
34351at9443	HAUS augmin-like complex subunit 7
29773at9443	spermatogenic leucine zipper protein 1
37395at9443	Prostaglandin E receptor 2
50487at9443	C-X-C motif chemokine
47105at9443	MRG domain binding protein
48788at9443	nuclear transition protein 2
36159at9443	myogenic differentiation 1
12936at9443	Hermansky-Pudlak syndrome 6 protein
33785at9443	Zinc finger HIT-type containing 2
45736at9443	Inhibitor of DNA binding 2
32896at9443	Fos proto-oncogene, AP-1 transcription factor subunit
51006at9443	C-X-C motif chemokine ligand 17
6136at9443	Catsper channel auxiliary subunit epsilon
21035at9443	Porcupine O-acyltransferase
31347at9443	Developing brain homeobox 2
33487at9443	coiled-coil domain-containing protein 185
30663at9443	XK related 8
26532at9443	LOW QUALITY PROTEIN: DEP domain-containing protein 4
50689at9443	Guanylate cyclase activator 2A
51648at9443	Fc fragment of IgE receptor Ig
50703at9443	COMM domain containing 6
36587at9443	F-box protein 45
39130at9443	Follistatin like 3
9372at9443	ERCC excision repair 2, TFIIH core complex helicase subunit
24891at9443	Neuronal pentraxin 2
22847at9443	Cholinergic receptor nicotinic alpha 1 subunit
43560at9443	Heat shock protein family B (small) member 8
37096at9443	Limb and CNS expressed 1
44197at9443	Protein lin-7 homolog
24031at9443	Chromosome 11 open reading frame 95
46503at9443	Claudin
42640at9443	Transmembrane BAX inhibitor motif containing 4
34523at9443	GLIPR1 like 2
20110at9443	SAMM50 sorting and assembly machinery component
41928at9443	Fibroblast growth factor
31034at9443	IKBKB interacting protein
42371at9443	Heme binding protein 1
37440at9443	Leucine rich repeat containing 73
27254at9443	retrotransposon Gag like 3
34000at9443	BCL2 associated athanogene 4
49371at9443	Coiled-coil-helix-coiled-coil-helix domain containing 1
14303at9443	Acyl-CoA dehydrogenase very long chain
44991at9443	Coiled-coil domain containing 184
47670at9443	charged multivesicular body protein 4b
39093at9443	PGAM family member 5, mitochondrial serine/threonine protein phosphatase
28719at9443	zinc finger protein 296
23559at9443	protein Red
44208at9443	Cerebellin 1 precursor
46000at9443	regulator of G-protein signaling 21
12690at9443	Sirtuin 1
49217at9443	somatostatin
40558at9443	Glycoprotein A33
48167at9443	Chromosome 11 open reading frame 58
8444at9443	Hepatocyte growth factor
22040at9443	Mitochondrial import inner membrane translocase subunit TIM44
24327at9443	Tachykinin receptor 2
38609at9443	Cytochrome b reductase 1
12036at9443	Carnitine palmitoyltransferase 2
23394at9443	Unc-51 like kinase 3
35215at9443	Caveolae associated protein 1
19175at9443	F-box only protein 33
40743at9443	Chromosome 6 open reading frame 229
27103at9443	probable G-protein coupled receptor 152
47380at9443	Transmembrane protein 114
26966at9443	5-hydroxytryptamine receptor 1D
13341at9443	transcription initiation factor TFIID subunit 5
30524at9443	Aquaporin 4
40672at9443	TIMP metallopeptidase inhibitor 4
48201at9443	natriuretic peptides A
40107at9443	Leucine rich repeat containing 38
28606at9443	E2F transcription factor 2
19164at9443	Aspartyl-tRNA synthetase
2190at9443	intron-binding protein aquarius
33381at9443	Family with sequence similarity 217 member B
41995at9443	rho GDP-dissociation inhibitor 3
45264at9443	VPS37B, ESCRT-I subunit
41933at9443	Ephrin A1
47322at9443	Cysteine rich hydrophobic domain 2
26709at9443	Adrenoceptor alpha 2A
7275at9443	adhesion G-protein coupled receptor D2
48150at9443	interleukin-9
29163at9443	Alpha-1-microglobulin/bikunin precursor
5423at9443	Mtr4 exosome RNA helicase
24084at9443	Ubiquilin 1
39490at9443	Family with sequence similarity 180 member B
42003at9443	Peroxisomal biogenesis factor 11 gamma
38928at9443	MKRN2 opposite strand protein
43116at9443	Potassium channel tetramerization domain containing 2
37680at9443	BMP and activin membrane-bound inhibitor homolog
26412at9443	bystin
37942at9443	estradiol 17-beta-dehydrogenase 8
17177at9443	nuclear receptor subfamily 1 group D member 2
24897at9443	complement C1q tumor necrosis factor-related protein 3 isoform X1
43445at9443	RAS related
38510at9443	fragile X mental retardation 1 neighbor protein
32667at9443	coiled-coil domain-containing protein 54
23545at9443	Acetyl-CoA acetyltransferase 2
31529at9443	Geminin coiled-coil domain containing
37780at9443	Zinc finger protein 688
39857at9443	collagen alpha-1(XXIII) chain
21645at9443	lamin-B1
44035at9443	NADH
38186at9443	Visual system homeobox 1
30107at9443	torsin-1A
47192at9443	Interleukin 17A

48659at9443	EP300-interacting inhibitor of differentiation 2
31645at9443	E2F transcription factor 4
32598at9443	Leucine rich repeat containing 52
26873at9443	Cartilage associated protein
39678at9443	Intercellular adhesion molecule 2
13013at9443	retinitis pigmentosa 1-like 1 protein
33879at9443	protein FAM228B
29068at9443	Acrosin
37388at9443	Homeobox C13
24555at9443	5-hydroxytryptamine receptor 3B
44835at9443	BLOC-1 related complex subunit 6
48408at9443	myotrophin
34336at9443	Biphenyl hydrolase like
17803at9443	Heparanase
36872at9443	deoxycytidine kinase
30156at9443	Caveolae associated protein 4
43903at9443	Peptidyl-prolyl cis-trans isomerase
22509at9443	growth hormone-releasing hormone receptor
19187at9443	carboxylesterase 4A
29047at9443	G-protein coupled receptor 15
18053at9443	Alkaline phosphatase
25476at9443	diphthamide biosynthesis 1
29486at9443	dentin sialophosphoprotein
46267at9443	Cornichon family AMPA receptor auxiliary protein 2
38243at9443	phenylethanolamine N-methyltransferase
28474at9443	Aminocarboxymuconate semialdehyde decarboxylase
26176at9443	Coagulation factor X
45586at9443	phospholipase A2 group IIE
34526at9443	Rab interacting lysosomal protein
13849at9443	Syntaxin binding protein 3
30495at9443	Angiopoietin like 7
35918at9443	olfactory receptor 52W1
38333at9443	ELL-associated factor 1
30814at9443	Trace amine associated receptor 5
42624at9443	SPRY domain containing 4
28637at9443	gastrin-releasing peptide receptor
24106at9443	forkhead box protein C2
38640at9443	Mitochondrial ribosomal protein L16
37907at9443	Centromere protein R
28202at9443	TNFAIP3 interacting protein 2
21438at9443	carboxypeptidase A6
44880at9443	Apolipoprotein D
2777at9443	maestro heat-like repeat family member 5
36039at9443	Proteasome assembly chaperone 1
50200at9443	Transmembrane protein 233
45302at9443	Peptidyl-prolyl cis-trans isomerase
33708at9443	Ephrin B1
15696at9443	tRNA wybutosine-synthesizing protein 4
36607at9443	Mitochondrial ribosomal protein L46
42958at9443	C-type lectin domain family 3 member B
16622at9443	Ankyrin repeat and ubiquitin domain containing 1
33612at9443	Testis expressed 26
36652at9443	forkhead box protein B1
41690at9443	Homeobox B5
15895at9443	catalase
28239at9443	Transcobalamin 1
1196at9443	CREB binding protein
36333at9443	Dehydrogenase/reductase 13
36680at9443	DNA-directed RNA polymerase II subunit RPB3
25782at9443	ankyrin repeat and SOCS box protein 18
48770at9443	N-acetyltransferase domain containing 1
43787at9443	Claudin
26844at9443	Tachykinin receptor 3
25503at9443	Arrestin domain containing 4
35252at9443	homeobox protein aristaless-like 3
11453at9443	acyl-CoA oxidase 1
14822at9443	Oncoprotein induced transcript 3
40324at9443	Forkhead box A3
25027at9443	Cell cycle checkpoint control protein
24679at9443	Potassium voltage-gated channel subfamily A member 6
38919at9443	taste receptor type 2 member 16
19158at9443	Patatin like phospholipase domain containing 3
39504at9443	Neurexophilin
31447at9443	G protein-coupled receptor 137C
36769at9443	NADH
37487at9443	SLAM family member 6
42792at9443	transmembrane protein 247
45251at9443	Transmembrane 4 L six family member 4
27145at9443	DDB1- and CUL4-associated factor 7
5154at9443	nestin
26345at9443	chloride intracellular channel protein 6
31879at9443	ADP-ribosylhydrolase like 2
24415at9443	Family with sequence similarity 71 member B
38325at9443	Asialoglycoprotein receptor 1
23292at9443	WD repeat and SOCS box containing 2
48099at9443	Mitochondrial ribosomal protein S14
43257at9443	homeobox protein engrailed-2
23219at9443	PHD finger protein 21B
49223at9443	Prokineticin 2
28858at9443	Queuosine salvage protein
41794at9443	Phospholipase A2 group XIIB
43100at9443	Nucleoredoxin like 1
41659at9443	mas-related G-protein coupled receptor member G
28079at9443	ethanolaminephosphotransferase 1
44773at9443	neurogenin-1
30916at9443	Forkhead box I1
27754at9443	carbohydrate sulfotransferase 14
38561at9443	Homeobox B8
11015at9443	Actin filament associated protein 1 like 1
39576at9443	Chromosome 3 open reading frame 70
35586at9443	Neuropeptides B and W receptor 1
37439at9443	Olfactory receptor
38796at9443	osteopetrosis-associated transmembrane protein 1
36814at9443	carboxymethylenebutenolidase homolog
19283at9443	Muscarinic acetylcholine receptor
49816at9443	Succinate dehydrogenase complex assembly factor 4
32887at9443	Leucine rich alpha-2-glycoprotein 1
7882at9443	integrator complex subunit 5
36743at9443	serine protease 27
38129at9443	Transmembrane protein 38A
29179at9443	ceramide synthase 5 isoform X1
37074at9443	Ankyrin repeat domain 60
31514at9443	Cathepsin S
40102at9443	Kallikrein related peptidase 4

3426at9443	Fibulin 2
39962at9443	Barttin CLCNK type accessory beta subunit
23600at9443	Coiled-coil domain containing 105
31941at9443	TNFAIP3-interacting protein 3
37072at9443	Coiled-coil domain containing 189
38833at9443	Transmembrane protein 53
22385at9443	TNF receptor superfamily member 11b
12383at9443	synaptonemal complex protein 2-like
42458at9443	Homeobox C4
38973at9443	Developing brain homeobox 1
28588at9443	Armadillo repeat containing 12
12071at9443	Arachidonate 15-lipoxygenase, type B
35007at9443	Sideroflexin
40156at9443	Nudix hydrolase 8
42202at9443	RAB22A, member RAS oncogene family
37825at9443	TatD DNase domain containing 3
19192at9443	prostacyclin synthase
29483at9443	Cholecystokinin B receptor
40879at9443	Receptor transporter protein 3
45038at9443	Transmembrane 4 L six family member 5
33963at9443	testis, prostate and placenta expressed
32311at9443	coiled-coil domain-containing protein 96
36346at9443	Cerberus 1, DAN family BMP antagonist
50815at9443	C-C motif chemokine
20770at9443	Cytochrome P450 family 27 subfamily A member 1
32912at9443	GPN-loop GTPase 2
40872at9443	glutathione peroxidase 3
49029at9443	Shisa like 2B
9072at9443	Chromosome 9 open reading frame 131
15615at9443	Oxidative stress induced growth inhibitor family member 2
15970at9443	Methyltransferase like 3
48301at9443	Protein phosphatase 1 regulatory subunit 27
47507at9443	endothelial cell-specific chemotaxis regulator
7923at9443	DNA replication licensing factor MCM6
19606at9443	Acrosin binding protein
28722at9443	peroxisomal biogenesis factor 7
36960at9443	aquaporin-1
33208at9443	Cyclin N-terminal domain containing 1
35781at9443	Olfactory receptor
51464at9443	tumor necrosis factor receptor superfamily member 13C
17525at9443	DNA-(apurinic or apyrimidinic site) lyase
14214at9443	Kelch like family member 21
18473at9443	Matrix metallopeptidase 21
42321at9443	Inhibitor of growth protein
43933at9443	PRA1 family protein
45445at9443	putative claudin-25
48757at9443	Complexin 4
38137at9443	Homeobox D1
9311at9443	NOP2/Sun RNA methyltransferase family member 2
6465at9443	DNA mismatch repair protein
27980at9443	Cytidine/uridine monophosphate kinase 2
44721at9443	Myosin light chain, phosphorylatable, fast skeletal muscle
1897at9443	ATP binding cassette subfamily A member 3
45156at9443	Cerebellin 4 precursor
26456at9443	Transmembrane 6 superfamily member 2
42417at9443	Pancreatic and duodenal homeobox 1
18662at9443	Tigger transposable element derived 5
21633at9443	UBX domain protein 11
41538at9443	keratin-associated protein 27-1
10023at9443	Cyclin F
40749at9443	Pleckstrin homology domain containing B1
48346at9443	apolipoprotein C-IV
35928at9443	C-X-C motif chemokine receptor 3
41575at9443	testis expressed 35
28119at9443	Four jointed box 1
50948at9443	Regulator of cell cycle
26351at9443	calreticulin-3
44080at9443	Glial cell derived neurotrophic factor
6637at9443	DDHD domain containing 1
26162at9443	Receptor for activated C kinase 1
35300at9443	Hydroxysteroid 17-beta dehydrogenase 3
13764at9443	Collagen type IV alpha 1 chain
25631at9443	Mex-3 RNA binding family member B
39033at9443	homeobox protein orthopedia
39241at9443	TIMP metallopeptidase inhibitor 3
15395at9443	ankyrin repeat and death domain containing 1A
51220at9443	protein S100-P
27374at9443	WD repeat domain 73
20642at9443	Ubiquinol-cytochrome c reductase core protein 1
49763at9443	Glutaredoxin 5
25524at9443	Tryptophan 2,3-dioxygenase
34783at9443	Aspartate dehydrogenase domain containing
16791at9443	Acid sensing ion channel subunit family member 4
48919at9443	Urotensin 2
6294at9443	melanoma-associated antigen E1
38702at9443	taste receptor type 2 member 4
19451at9443	Drebrin 1
22095at9443	APC down-regulated 1 like
46904at9443	Regulator of G protein signaling 9 binding protein
37086at9443	voltage-dependent calcium channel gamma-8 subunit
39616at9443	Homeobox B2
38016at9443	Transmembrane protein 64
46212at9443	neuroglobin
24946at9443	Acid phosphatase, prostate
7810at9443	Fc receptor like 5
43232at9443	Crystallin beta A2
39902at9443	Fibroblast growth factor
46520at9443	heat shock protein beta-3
34276at9443	Insulin like growth factor binding protein 2
36243at9443	Proline rich 32
40521at9443	splicing factor 3B subunit 4
18907at9443	platelet glycoprotein V
41763at9443	Peptidyl-prolyl cis-trans isomerase
27245at9443	exosome complex component RRP45
44022at9443	ventral anterior homeobox 2
41679at9443	Sjogren syndrome/scleroderma autoantigen 1
47619at9443	Fer3 like bHLH transcription factor
10074at9443	Arachidonate 12-lipoxygenase, 12R type
42683at9443	Protein tyrosine phosphatase receptor type C-associated protein
25900at9443	SET and MYND domain containing 2
24916at9443	Fc receptor like 1
24850at9443	meiotic recombination protein SPO11 isoform X1
19543at9443	nuclear receptor subfamily 0 group B member 1
14094at9443	DBF4 zinc finger

37017at9443	dopamine receptor D4
39839at9443	Spermatogenesis associated 9
25929at9443	Centromere protein T
44750at9443	chromosome 17 open reading frame 107
45686at9443	transmembrane protein 47
47189at9443	Endonuclease G
47536at9443	Troponin C2, fast skeletal type
25213at9443	Cell cycle control protein
13799at9443	Amyloid beta precursor like protein 2
12867at9443	Extracellular matrix protein 2
18237at9443	Protein disulfide isomerase family A member 5
29428at9443	Pannexin
12330at9443	Polypeptide N-acetylgalactosaminyltransferase
25931at9443	Abhydrolase domain containing 12B
26418at9443	GRINL1A complex locus 1
44858at9443	Kruppel like factor 14
38361at9443	TATA-box binding protein associated factor 9b
15844at9443	Tyrosine-protein kinase
4099at9443	Ceruloplasmin
20973at9443	Essential meiotic structure-specific endonuclease 1
50229at9443	COX19, cytochrome c oxidase assembly factor
17829at9443	Mitochondrial elongation factor 2
33977at9443	Ribonuclease H2 subunit B
39559at9443	glutathione peroxidase 6
26827at9443	Sorting nexin
45243at9443	protein jagunal homolog 1
27098at9443	Adrenoceptor alpha 2B
39292at9443	Rho family GTPase 1
46711at9443	Epithelial mitogen
50850at9443	Galanin and GMAP prepropeptide
34927at9443	Potassium channel tetramerization domain containing 12
9249at9443	AP-1 complex subunit gamma
32377at9443	Elongation factor Ts, mitochondrial
17220at9443	Activating transcription factor 6 beta
42698at9443	Shisa like 2A
16409at9443	Leucine rich repeats and calponin homology domain containing 4
26075at9443	Abhydrolase domain containing 15
42039at9443	N-6 adenine-specific DNA methyltransferase 1
20855at9443	Ectonucleoside triphosphate diphosphohydrolase 6 (putative)
27409at9443	Protein Wnt
40903at9443	Tissue factor
29482at9443	Plasmalemma vesicle associated protein
14239at9443	dopamine beta-hydroxylase
19835at9443	negative elongation factor B
38608at9443	Radial spoke head 9 homolog
31476at9443	TEA domain transcription factor 4
37967at9443	transmembrane emp24 domain-containing protein 6
43938at9443	Ladybird homeobox 2
32569at9443	S-methyl-5'-thioadenosine phosphorylase
23735at9443	CCR4-NOT transcription complex subunit 11
10820at9443	Sulfhydryl oxidase
8709at9443	aryl hydrocarbon receptor
29677at9443	Paraoxonase 3
21101at9443	tRNA wybutosine-synthesizing protein 2 homolog
29843at9443	protein FAM117B
29153at9443	Elongation of very long chain fatty acids protein 2
23970at9443	Glucagon receptor
31003at9443	OTU deubiquitinase 1
31937at9443	C-X-C motif chemokine receptor 5
43597at9443	Interleukin 17C
10518at9443	ArfGAP with coiled-coil, ankyrin repeat and PH domains 3
19163at9443	zinc finger protein 689
27307at9443	Coagulation factor II thrombin receptor like 2
45516at9443	WASH complex subunit 3
41155at9443	Cysteine dioxygenase type 1
24531at9443	C-type lectin domain containing 14A
12528at9443	CD248 molecule
17432at9443	G protein-coupled receptor 107
10424at9443	Acyloxyacyl hydrolase
35245at9443	olfactory receptor 51G2
45348at9443	Mitochondrial ribosomal protein S25
21823at9443	lectin, mannose binding 1 like
50907at9443	28 kDa heat- and acid-stable phosphoprotein
28004at9443	ST6 N-acetylgalactosaminide alpha-2,6-sialyltransferase 2
41363at9443	Anti-silencing function 1A histone chaperone
27320at9443	Bombesin receptor subtype 3
43952at9443	Insulin like growth factor binding protein 6
33350at9443	Mitochondrial glycine transporter
51164at9443	C-C motif chemokine
28479at9443	Cell cycle control protein
42149at9443	Gap junction protein
30239at9443	Talin rod domain containing 1
35891at9443	chymotrypsin-like elastase family member 1
6474at9443	THAP domain containing 9
23940at9443	SH2 domain-containing adapter protein B
42790at9443	myogenic factor 6
38042at9443	zinc finger protein 784
26383at9443	Galactose-1-phosphate uridylyltransferase
50642at9443	GTP cyclohydrolase I feedback regulator
43494at9443	mitochondrial assembly of ribosomal large subunit protein 1
28983at9443	Layilin
28765at9443	MLLT3, super elongation complex subunit
10509at9443	TSR1, ribosome maturation factor
24232at9443	Acid phosphatase 2, lysosomal
10788at9443	Spermatogenesis associated 5 like 1
6093at9443	Spalt like transcription factor 4
23252at9443	Serum amyloid A like 1
39072at9443	JunB proto-oncogene, AP-1 transcription factor subunit
3435at9443	Valosin containing protein interacting protein 1
18213at9443	G protein-coupled receptor kinase
27011at9443	GDNF family receptor alpha-3
26033at9443	Four and a half LIM domains 1
43565at9443	transmembrane protein 223
29797at9443	Protein phosphatase 1 regulatory subunit
34562at9443	keratin 9
51454at9443	small integral membrane protein 13
50715at9443	Small leucine rich protein 1
45363at9443	Chromosome 1 open reading frame 185
39752at9443	Tyrosine 3-monooxygenase/tryptophan 5-monooxygenase activation protein gamma
30428at9443	E2F transcription factor 1
36758at9443	Zinc finger FYVE-type containing 21
16164at9443	Pleckstrin homology domain containing N1
2950at9443	Carboxypeptidase D
4362at9443	Leucine rich repeats and immunoglobulin like domains 3
36211at9443	Nicotinate-nucleotide pyrophosphorylase
22721at9443	major centromere autoantigen B
43678at9443	signal peptidase complex subunit 3
37185at9443	Glutamate-cysteine ligase modifier subunit
22943at9443	Mannosyltransferase
5354at9443	RAD51-associated protein 2
26304at9443	Transmembrane 7 superfamily member 2
28208at9443	Transmembrane and coiled-coil domains 6
11069at9443	Zinc finger protein 792
37169at9443	probable threonine protease PRSS50
16806at9443	Sorting nexin
43679at9443	Calmodulin like 6
37699at9443	Homeobox A11
44609at9443	Histidine triad nucleotide binding protein 3
24983at9443	Keratin 74
44243at9443	transmembrane and coiled-coil domain-containing protein 2
44736at9443	peptidoglycan recognition protein 1
42340at9443	Cyclin dependent kinase inhibitor 1B
48103at9443	Fatty acid binding protein 2
35078at9443	olfactory receptor 10AD1
49583at9443	DR1 associated protein 1
41474at9443	Shisa family member 3
37860at9443	Polycomb group ring finger 1
42506at9443	BRICHOS domain containing 5
40263at9443	SIX homeobox 6
26504at9443	solute carrier family 49 member 3
47920at9443	Transthyretin
38731at9443	Insulin like growth factor binding protein 4
43841at9443	Brain specific homeobox
40171at9443	replication protein A 30 kDa subunit
21363at9443	Docking protein 1
11643at9443	Ribosome biogenesis protein BOP1
7366at9443	Nuclear factor of activated T cells 2
39851at9443	SLAM family member 8
10128at9443	Trichohyalin like 1
38029at9443	BARX homeobox 2
18569at9443	Aldehyde dehydrogenase 8 family member A1
22918at9443	glucagon-like peptide 1 receptor
29218at9443	Protein Wnt
36723at9443	calbindin
34725at9443	LysM domain containing 3
22511at9443	Zinc finger protein 514
32733at9443	ankyrin repeat and SOCS box protein 17
43287at9443	RING finger protein 186
34696at9443	Beta-1,3-N-acetylglucosaminyltransferase
25548at9443	Cytochrome P450 family 2 subfamily S member 1
41184at9443	RAS like family 11 member B
9669at9443	Tripartite motif containing 71
36135at9443	MAD2L1 binding protein
45965at9443	dCTP pyrophosphatase 1
10614at9443	Chloride channel protein
18643at9443	MAGE family member E2
19763at9443	Matrix metallopeptidase 19
33752at9443	NFKB inhibitor alpha
40502at9443	regulated endocrine-specific protein 18
23730at9443	DnaJ heat shock protein family (Hsp40) member A3
10389at9443	Death domain containing 1
27438at9443	Podocalyxin
38802at9443	Nudix hydrolase 21
31986at9443	Stanniocalcin 2
22776at9443	HtrA serine peptidase 1
47434at9443	RING finger protein 11
25950at9443	Growth differentiation factor 6
32222at9443	Gamma-glutamyl hydrolase
26071at9443	Arrestin domain containing
